# List of reviewers

**DOI:** 10.2471/BLT.23.970123

**Published:** 2023-01-01

The Editorial Team would like to thank all those who gave generously of their time and expertise in reviewing the papers for the *Bulletin of the World Health Organization *in 2022. We apologize to anyone whose name has been inadvertently omitted from the following list:

## A

Abdullahi, Auwal

Abeykoon, Palitha

Abo-Rahma, Alyaa

Abramoff, Michael

Adair, Tim

Adekanmbi, Victor

Aduo-Adjei, Kofi

Aggarwal, Bhagwan

Ahmad, Aasim

Ahmad, Abdul

Ahmad, Irshad

Ahmat, Adam

Ajuebor, Onyema

Aklilu, Abenezer

Aksu Tanik, Feride

Akter, Tasnima

Alaama, Ahmed Sabry

Alexander, David

Allan, Jennifer

Allen, Koya

Allen, Matthew

Allipour Birgani, Ramesh

Aloosh, Mehdi

Amul, Gianna Gayle

Anaele, Beverly

Andualem, Tenaw

Anish, TS

Apisarnthanarak, Anucha

Arab-Zozani, Morteza

Armenta-Paulino, Nancy

Armstrong, Kirsten

Asokan, G

Andreas Ateke, Njoh

Auger, Nathalie

Awan, Haroon

Awasthi, Shally

Azmat, Syed Khurram

## B

Babu, Bontha

Baek, Yeji

Bahena, Alejandro

Bahl, Sunil

Baller, April

Barber, Sarah

Barnes, Karen

Barnhart, Dale

Baxi, Maulik

Beer, Tom

Begum, Shahnur

Bellingham-Young, Denise

Bergevin, Yves

Berhane, Yemane

Beynon, Fenella

Bhambere, Sailee

Bhandari, Nita

Birckmayer, Jo

Boeckmann, Melanie

Brieger, William

Briggs, Andrew

Brisson, Julien

Brown, Darren

Bruno, Alfredo

Bryant-Comstock, Katelyn

Butrick, Elizabeth

## C

Candeias, Vanessa

Cardona, Carolina

Carrasco-Labra, Alonso

Carregaro, Rodrigo

Carrizosa, Jaime

Case, Bruce

Castro Delgado, Rafael

Cecchini, Michele 

Chawla, P

Chhabra, Pragti

Chippaux, Jean-Philippe

Chiwaula, Levison

Cieza, Alarcos

Ciptaningtyas, Ratri

Citron, Isabelle

Clark, Jackie

Clark, Kathleen

Codyre, Patricia

Cook, Margaret

Crainey, James

Cress, Cynthia

Cunningham, Nicola

## D

Dartnall, Elizabeth

Dary, Omar

Das, Saibal

de Haas, Billie

de Witte, Luc

Debes, Jose

Deboutte, Danielle

Do, Mai

Doke, Prakash

Downs, Shauna

Durkin, Maureen

## E

Edward, Anbrasi

Eikelboom, Robert

Einarsdóttir, Kristjana

El-Osta, Austen

Engelhardt, Katrin

## F

Fagan, Johannes

Feelemyer, Jonathan

Feng, Shanwei

Feng, Wenli

Feng, Xing Lin

Fenny, Ama

Fernandes, Edmond

Ferrecio, Caterina

Ferri de Barros, Fábio

Fisher, Jane

Fitzpatrick, Siobhan

Fleischer, Angelica

Ford, Nathan

Fraser, Joy

Freak-Poli, Rosanne

Frontera, Walter

## G

Gabani, Jacopo

Garrison, Karma

Gaule, Patrick

Gautham, Meenakshi

Ge, Yang

Ghosh, Kanjaksha

Gill, Christopher

Gimbel, Sarah

Giref, Ababi

Godfrey, Katy

Godman, Brian

Goodchild, Mark

Gorina, Yelena

Graham, Stephen

Greening, Bradford

Grills, Nathan

Gunawardane, Damitha

## H

Hajarizadeh, Behzad

Harrison, Alexandra

Heidari, Shirin

Heinmüller, Rolf

Henn, Wolfram

Hermoso de Mendoza, Javier

Ho, Li-Li

Holmgren, Christopher

Houghton, Adele

Hu, Guoqing

Husain, Sara

Hussein, Ghaiath

## I

Ide, Nicole

Ikenyei, Uche

Imi, Monica

Inyang, Ibanga

## J

Jacdonmi, Itse

Jassat, Waasila

Jin, Zi-Bing

Jindal, Rahul

Johns, Timothy

Johnson, Mark

Johnston, Brian

## K

Kaur, Jagdish

Keenan, Jeremy

Kemmanu, Vasudha

Kharbanda, Om

Khayesi, Meleckidzedeck

Khosravi, Ardeshir

Kinder, Karen

King, Jessica

Kinney, Mary

Knight, Gwenan

Kosen, Soewarta

Kumar, Pratap

Kumaravel, Shanmugasundaram

Kwamie, Aku

## L

Labonte, Ron

Lackland, Daniel

Lakshmi, Josyula

Lantagne, Daniele

Lau, Eric

Lau, Evan

Lazarus, Jeffrey

Lee, Lois

Lekagul, Angkana

Lencucha, Raphael

Lengeler, Christian

Lin, Yan

Lindner, Andre

Liu, Jifeng

Liu, Zhiyong

Llewellyn, Gwynnyth

Löfmark, Sonja

Lotfizadeh, Masoud

Louw, Quinette

Lozano, Juan

Lumpkin, Murray

Lunt, Neil

Luschin-Ebengreuth, Marion

Luyckx, Valerie

## M

Machalaba, Catherine

Macharia, Peter

MacLean, David

Maheshwar, P K

Maibach, Edward

Maina, Mercy

Malqvist, Mats

Mangadan-Konath, Nabeel

Mao, Wenhui

Marrugo-Negrete, José

Martinez, Rodrigo 

Mason, Ingrid

Masood, Fakeha

Mathai, Mathews

Mathews, Priya

Matsuyama, Yusuke

Mavrou, Katerina

McBain, Ryan

McMahon, Graham

Mehraj, Vikram

Melberg, Andrea

Mentis, Alexios-Fotios

Mileder, Lukas

Miller, Lewis

Mills, Jody-Anne

Moitinho de Almeida, Maria

Moja, Lorenzo

Montgomery, Maggie

Mossey, Peter

Mugabo Semahore, Jules

Mugambe, Richard

## N

Nakayama, Luis

Narayan, Choudhary

Naumova, Elena

Nkangu, Miriam

Noel, Jonathan

Nola, Iskra

Noureen, Sibgha

Nurcahyanti, Agustina

## O

O'Hara, Lyndsay

Oktaria, Vicka

Olusanya, Bolajoko

Ortega, Francisco

Osibogun, Olatokunbo

## P

Pachman, Joseph

Paichadze, Nino

Pan, Jay

Papadopoulou, Athina

Pardosi, Jerico

Pate, Russell

Pathak, Rambha

Paul, Bobby

Picchio, Camila

Pollack, Keshia

Poppe, Olav

Prasertchai, Araya

Pu, Christy

Puri, Parul

## Q

Queiroz, Bernardo

## R

Rackimuthu, Sudhan

Raghunath Rao, Rangasayee

Rai, Saraswathi Bina

Ramanathan, Mala

Ranjan, Alok

Ravichandran, Natraj

Reacher, Mark

Read, Jennifer

Riboulet-Zemouli, Kenzi

Richards, Nicola

Risal, Ajay

Roberts, Sarah

Roberts, Tessa

Robinson, David

Rodewald, Lance

Rodriguez, Francisca

Rodriguez, Natalia

Rodriguez-Bano, Jesus

Rüegg, Simon

Rupp, Bernhard

## S

Saksena, Nivedita

Samuels, Jay

Sayed, Shaheen

Schaaf, Marta

Schmidt, Elena

Schuftan, Claudio

Scott, Susana

Seymour, Brittany

Shankar, Bhavani

Sharland, Mike

Shillcutt, Samuel

Shridhar, Manisha

Silenou, Bernard Chawo

Silva, Maria-Raquel G

Sinclair, Kit

Singh, Gagandeep

Sly, Peter

Smith, Andrew

Smith, Julie

Smith, Rebecca

Solomon, Anthony

Song, Liting

Soudarssanane, M Bala

Soyiri, Ireneous

Standl, Fabian

Stoeckl, Heidi

Stowell, Daniel

Stuckler, David

Sturchio, Jeffrey

Sultana, Papia

Sun, Qiang

Suthar, Amitabh

Sy, Deborah

## T

Tagboto, Senyo

Tan, Kok-Chor

Tandale, Babasaheb

Tandoc III, Amado

Tang, Yi

Tayler, Elizabeth

Teboh-Ewungkem, Miranda

Terrones-Lozano, Alejandro

Thompson, Michael

Tibig, Lourdes

Tomasoni, Giuseppe

Tort, Julie

Triep, Karen

Vashishtha, Kriti

## V

Villa, Simone

Villalba, Julian

Vinikoor, Michael

Visekruna Vucina, Vesna

Vogler, Sabine

Vokinger, Kerstin N

## W

Wagley, Ujjal

Wagner, Claire

Wang, Chao

Wang, Xiaomin

Ward, Kim

Watkins, David

Weerasuriya, Krisantha

Willcox, Merlin

Willetts, Elizabeth

Wilson, Nevin

Wu, Chenkai

## Y

Yang, Haomin

Yang, Li

Yansaneh, Aisha

Yew, Ying Ying

## Z

Zaeri-Isfahani, Amir Mahdi

Syed Hasan Zaidi, 

Zeitouny, Seraphine

Zhang, Chen

Zhang, Haijun

Zhang, Yuling

Zipursky, Simona

